# Morphological and digestive adjustments buffer performance: How staging shorebirds cope with severe food declines

**DOI:** 10.1002/ece3.5013

**Published:** 2019-03-12

**Authors:** Shou‐Dong Zhang, Zhijun Ma, Chi‐Yeung Choi, He‐Bo Peng, David S. Melville, Tian‐Tian Zhao, Qing‐Quan Bai, Wen‐Liang Liu, Ying‐Chi Chan, Jan A. van Gils, Theunis Piersma

**Affiliations:** ^1^ Ministry of Education Key Laboratory for Biodiversity Science and Ecological Engineering, Coastal Ecosystems Research Station of the Yangtze River Estuary, Shanghai Institute of Eco‐Chongming (SIEC) Fudan University Shanghai China; ^2^ Department of Coastal Systems, NIOZ Royal Netherlands Institute for Sea Research and Utrecht University Texel The Netherlands; ^3^ Rudi Drent Chair in Global Flyway Ecology, Conservation Ecology Group, Groningen Institute for Evolutionary Life Sciences (GELIFES) University of Groningen Groningen The Netherlands; ^4^ School of Biological Sciences University of Queensland Brisbane Queensland Australia; ^5^ Centre for Integrative Ecology, School of Life & Environmental Sciences Deakin University Geelong Victoria Australia; ^6^ School of Environmental Science and Engineering Southern University of Science and Technology Shenzhen China; ^7^ Global Flyway Network Nelson New Zealand; ^8^ Forestry Bureau of Dandong Dandong China; ^9^ School of Ecological and Environmental Sciences East China Normal University Shanghai China

**Keywords:** East Asian‐Australasian Flyway, energetics, food decline, Great Knot *Calidris tenuirostris*, phenotypic flexibility, prey quality, regurgitates

## Abstract

Organisms cope with environmental stressors by behavioral, morphological, and physiological adjustments. Documentation of such adjustments in the wild provides information on the response space in nature and the extent to which behavioral and bodily adjustments lead to appropriate performance effects. Here we studied the morphological and digestive adjustments in a staging population of migrating Great Knots *Calidris tenuirostris* in response to stark declines in food abundance and quality at the Yalu Jiang estuarine wetland (northern Yellow Sea, China). At Yalu Jiang, from 2011 to 2017 the densities of intertidal mollusks, the food of Great Knots, declined 15‐fold. The staple prey of Great Knots shifted from the relatively soft‐shelled bivalve *Potamocorbula laevis* in 2011–2012 to harder‐shelled mollusks such as the gastropod *Umbonium thomasi* in 2016–2017. The crushing of the mollusks in the gizzard would require a threefold to 11‐fold increase in break force. This was partially resolved by a 15% increase in gizzard mass which would yield a 32% increase in shell processing capacity. The consumption of harder‐shelled mollusks was also accompanied by reliance on regurgitates to excrete unbreakable parts of prey, rather than the usual intestinal voidance of shell fragments as feces. Despite the changes in digestive morphology and strategy, there was still an 85% reduction in intake rate in 2016–2017 compared with 2011–2012. With these morphological and digestive adjustments, the Great Knots remaining faithful to the staging site to a certain extent buffered the disadvantageous effects of dramatic food declines. However, compensation was not complete. Locally, birds will have had to extend foraging time and use a greater daily foraging range. This study offers a perspective on how individual animals may mitigate the effects of environmental change by morphological and digestive strategies and the limits to the response space of long‐distance migrating shorebirds in the wild.

## 1 INTRODUCTION

When animals encounter problems in achieving a positive energy balance, they may move away (Piersma, 2012), adjust behavior (Sydeman et al., 2001), or change relevant morphological (Grant & Grant, 2017), and correlated physiological (i.e., “physiomorphic,” Oudman et al., 2016) aspects of the phenotype (Piersma & van Gils, 2011). Bodily adjustments are usually studied under controlled experimental conditions (Dekinga, Dietz, Koolhaas, & Piersma, 2001; van Gils, Piersma, Dekinga, & Dietz, 2003). Here we provide field evidence for phenotypic adjustment in a highly site‐faithful and long‐lived, long‐distance migratory shorebird species confronted with dramatic changes in their food supply at their main staging site.

Long‐distance migratory shorebirds have provided several landmark examples of phenotypic flexibility (Piersma, 2002), including extensive body remodeling during migration (Vézina, Williams, Piersma, & Morrison, 2012). During the staging episodes prior to departure on long‐distance flight, digestive and exercise organs grow and shrink in adaptive ways as the fuel stores are built up (Piersma & Gill, 1998; Piersma, Gudmundsson, & Lilliendahl, 1999). Of particular interest, here are the digestive organs. Shorebirds may regress their digestive organs (also some other visceral organs) in the course of long‐distance migratory flights (Battley et al., 2000), but need to upregulate them during fueling (Battley, Dekinga, et al., 2001; Battley, Dietz, et al., 2001; Battley et al., 2000; Landys‐Ciannelli, Piersma, & Jukema, 2003; Lindström & Piersma, 1993).

**Figure 1:**
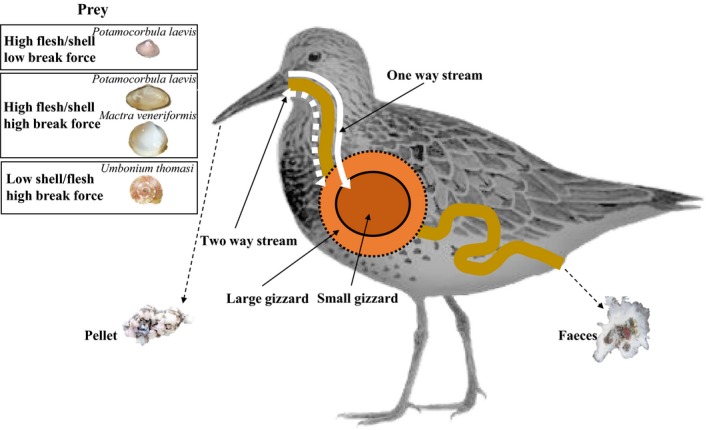
Summary of the factors contributing to different feeding performance of Great Knot. Depending on prey quality (flesh/shell ratio) and shell hardness (break force), Great Knots may have smaller or larger gizzards. Generally, crushed shells are evacuated through the gut as feces (one‐way stream), while Great Knots can also void unbreakable parts of ingested shells as pellets (two‐way stream)

We studied Great Knots *Calidris tenuirostris*, an endangered endemic shorebird species in the East Asian‐Australasian Flyway, seasonally commuting between main nonbreeding grounds in Northwest Australia and breeding grounds in the highlands of eastern Siberia, with halfway staging areas on the East Asian coast (Battley, Dekinga, et al., 2001; Battley, Dietz, et al., 2001; Battley et al., 2000; Lisovski, Gosbell, Hassell, & Minton, 2016; Ma et al., 2013). Except for their time on the tundra breeding grounds, Great Knots feed on mollusks on intertidal mudflats (Choi et al., 2017; Tulp & de Goeij, 1994). Shelled prey items are swallowed whole and crushed in the muscular gizzard, the shell fragments being evacuated from the gut as shell‐rich feces (one‐way stream, Figure 1). It takes longer to process hard‐shelled than soft‐shelled prey, and thus, the digestive bottleneck due to consumption of hard‐shelled prey will cause a decrease in food intake rate (van Gils, Battley, Piersma, & Drent, 2005; van Gils et al., 2003).

**Figure 2:**
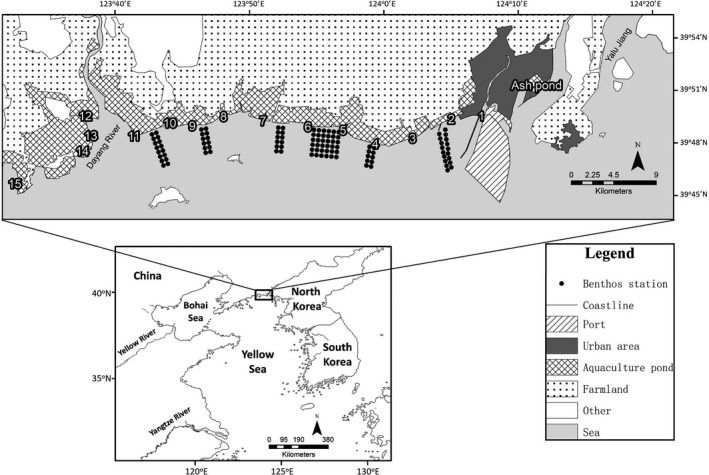
Location of Yalu Jiang estuarine wetland in the north part of the Yellow Sea, China. The dots show all the 104 macrobenthos stations sampled in 2013, 2015, 2016, and 2017. The 36 dots within the dashed‐line square in the central area show the macrobenthos stations sampled in 2011 and 2014; in 2012, we sampled the 36 sites within the dashed‐line square in the middle part together with the 12 sites within the dashed‐line rectangle in the east part

The decline in annual survival and population size of the Red Knot *Calidris canutus*, a sister species of the Great Knot, was earlier explained by food shortages at their main northward staging area due to overharvesting and low food availability at their wintering ground (Baker et al., 2004; van Gils et al., 2016). The recent declines in survival and population sizes of Great Knots have been explained by the loss and degradation of coastal staging habitat in the Yellow Sea region due to land claim, pollution, invasive species, and over‐exploitation of marine resources (Hua, Tan, Chen, & Ma, 2015; Melville, Chen, & Ma, 2016; Piersma et al., 2016). Following the destruction of Saemangeum, South Korea, by reclamation (Moores, Rogers, Rogers, & Hansbro, 2016), the Yalu Jiang estuarine wetland (hereafter YLJ, Figure 2) became the main known staging area of Great Knots in the Yellow Sea region (Choi, Battley, Potter, Rogers, & Ma, 2015; Riegen, Vaughan, & Rogers, 2014; Zhang et al., 2018). Despite there having been little loss of intertidal habitat over the past two decades at YLJ, the bivalve *Potamocorbula laevis*, one of the dominant benthic invertebrate species and the main prey of Great Knots (Choi et al., 2017), has dramatically declined in abundance since 2013 (Zhang et al., 2018).

Assessments of the degree to which such phenotypically flexible adjustments help animals in real‐life situations require field studies where the changing aspects of both the target species and their immediate environment including their food are described well enough (Battley, Dekinga, et al., 2001; Battley, Dietz, et al., 2001; Battley et al., 2000; Piersma, 2011). Here we assess how Great Knots staging at YLJ in 2011–2017 coped with the changing abundance and digestive quality of their food in terms of internal morphology and attributes of the digestion process (Figure 1). We analyzed food availability, composition, diet selection, and food intake rates in two years before and two years after the dramatic decline of *P. laevis*. We compared the quality of the different mollusk prey over the study period and examined changes in gizzard mass and the way that the birds coped with unbreakable parts of their prey. This study highlights how morphological and digestive strategies may change under resource limitation and offers a perspective on how a species can mitigate such effects (Figure 1).

## 2 MATERIALS AND METHODS

### 2.1 Study site

This study was carried out during March–May 2011–2017, at YLJ (39°40′–39°58′N, 123°34′–124°07′E; Figure 2), which is close to the national boundary between China and North Korea (Choi et al., 2017; Zhang et al., 2018). About 44,000 Great Knots (Zhang et al., 2018) stage here for nearly 2 months during northward migration (Choi et al., 2015; Ma et al., 2013; Riegen et al., 2014). From 2011–2012 to 2016–2017, the numbers of Great Knots at YLJ decreased by 29% (Zhang et al., 2018). The seasonal pattern of occurrence also changed. In the early years, numbers built up from late March to early April, to then remain steady until the time of northward departures from 14–21 May. However, departures substantially advanced in 2017, with numbers already declining from mid‐April. Some birds might move to other staging sites because it is too early to depart to the breeding grounds (Zhang et al., 2018). In 2015, a large number of Great Knots may have moved about 160 km westward from YLJ to Gaizhou, on the east of Liaodong Bay, Bohai (Melville, Peng, et al., 2016), where *P. laevis* were plentiful at the time (HBP and YCC unpublished data).

Tides at YLJ are typically semi‐diurnal, with an average tidal amplitude of 4.5 m and a maximum of 6.9 m (Wang, Ren, & Zhu, 1986). Local movements of Great Knots are strongly affected by the tides. They forage on the intertidal flats at low tide and roost in aquaculture ponds inside the seawall when intertidal flats are submerged (Zhang et al., 2018). Bivalves (especially superabundant *P. laevis*) have previously been identified as the staple food of Great Knots at YLJ (Choi et al., 2017).

### 2.2 Diet composition and food availability

To measure food availability, we sampled potential prey (macrobenthos, especially mollusks living in the soft sediments) once a month from March to May in 2011, 2012, 2015, 2016, and 2017, and April and May in 2013 and 2014. We established 16 sampling transects across the intertidal flats with a total of 104 sampling stations spaced 500 m apart (Figure 2). All the stations (104 sampling stations) were sampled in 2013, 2015, 2016, and 2017, but we focused on the middle parts (36 sampling stations) of the study area in 2011, 2012, and 2014 as this zone is the main foraging area of Great Knots (Choi et al., 2017). There is no significant difference in macrobenthos species and ash‐free dry mass (AFDM) between the middle parts of sampling stations and the total 104 sampling stations (*t* tests, *p* > 0.2 for both macrobenthos species and AFDM in all the years in 2013, 2015, 2016, and 2017) (Zhang et al., 2018). At each station, one core sample (diameter 15.5 cm, 5 cm in depth) was collected and then washed through a 0.5 mm sieve. We considered all bivalves and gastropods from the top 5 cm of sediment as potential food for Great Knots which have an average bill length of about 45 mm (Tulp & de Goeij, 1994). All soft‐bodied organisms were soaked in 5% formalin for at least 72 hr before being stored in 70% ethanol, and hard‐bodied organisms including large bivalves, gastropods, and crabs were kept frozen. Organisms were identified to the finest practicable taxonomic level (Supporting Information Table S1) with size (the longest measurement) measured (to 0.01 mm) in the laboratory (see Zhang et al., 2018). For each taxon, we selected complete individuals of different lengths to determine AFDM (Zhang et al., 2018).

We measured the height of the left hinge for different shell lengths of all bivalve species (Dekinga & Piersma, 1993; Yang et al., 2013), and the width of the last whorl of the columella for different sizes of *Umbonium thomasi*. The species‐specific relationships between size (the longest measurement) and AFDM, AFDM, and DM_shell_ (shell dry mass), size (the longest measurement) and height of left hinge or columella width were established using regression analysis (polynomial, e‐exponential, logarithmic, and power regression) for each taxonomic group (Bom et al., 2018). Models with the highest Pearson correlation coefficient were selected. Regression models were established for each year for the two dominant bivalves, *P. laevis* and *Mactra veneriformis*, while samples from all years were pooled for other groups in the regression models due to small sample sizes (Zhang et al., 2018).

Food retention time in the digestive tracts of sandpipers is short (e.g., 20–50 min for red knots, Piersma, 1994). During low tide, we followed flocks of Great Knot at foraging sites to collect droppings after birds had been feeding at a site for more than 30 min (to ensure the droppings were produced by Great Knots feeding on prey from that foraging site) (Choi et al., 2017). We collected a total of 2,712 droppings (1,093 in 2011, 856 in 2012, 117 in 2016 and 646 in 2017) and 439 pellets (184 in 2016 and 255 in 2017). Each dropping and pellet were placed in a separate plastic bag and stored at –20°C.

In the laboratory, droppings and pellets were dried at 60°C for 72 hr, and then sifted through a 0.3 mm sieve. The shell fragments from the droppings and pellets were sorted to species and measured to the nearest 0.1 mm using an Olympus SZX7 dissecting microscope: the height of unbroken hinges for bivalve species, the width of the last whorl of the columella for *U. thomasi*, and the shell length for undigested individuals. The weight of shell fragments (no columella present) and the columella of *U. thomasi *in droppings and pellets were weighed to the nearest 0.1 mg. To determine the size composition of ingested organisms contained in the dropping and pellet samples, we used the regression of left hinge height against shell length for bivalve species, and the regression between columella width and total width in *U. thomasi* (Supporting Information Table S3).

At YLJ, Great Knots mainly consumed *P. laevis*, but also took other bivalves and gastropods, all available prey being within the top 5 cm of sediment (Choi et al., 2017). Based on the analysis of feces and pellets, the maximum sizes of prey taken by Great Knots were determined (Zwarts & Blomert, 1992). We defined potential (“harvestable”) food as follows: *P. laevis* (less than 25 mm, divided into small size: <10 mm and large size: >10 mm) and *Moerella iridescens* less than 25 mm, *M. veneriformis* less than 23 mm, *U. thomasi *less than 15 mm, *Nassarius variciferus* and *Nassarius festivus* less than 12 mm, other smaller proportions of bivalves and gastropods less than 20 mm.

### 2.3 Intake rate, prey quality, and shell hardness

To record bird behavior, a focal bird was chosen randomly from a flock of foraging birds and watched for 5‐min, using a ×20–60 telescope. Before the start of each 5‐min observation bout, the date, time, and location were noted. During each observation bout, activities such as pecks, probes, items swallowed and interference with other individuals were recorded on digital voice recorders (2011 and 2012), while we used digital video cameras to record all behaviors in 2016 and 2017. The digital sound files were transcribed using JWatcher 1.0 (Blumstein, Daniel, & Evans, 2006), which allowed us to quantify the time a bird spent on different activities. We used BORIS (Friard & Gamba, 2016) to transcribe behaviors on video. We identified ingested prey as bivalve, crab, gastropod, ghost shrimp, razor clam, polychaeta, sea anemone, or unknown.

Based on the composition of the prey size and the regression of size‐species AFDM/DM_shell_ (Supporting Information Tables S1 and S2), we estimated ingested AFDM and DM_shell_ for Great Knots feeding on a specific food item. Based on the species and numbers of prey taken by Great Knots, and the relationship of size‐species AFDM/DM_shell_ (Supporting Information Tables S1 and S2), we calculated AFDM/DM_shell_ intake rate per unit time. We evaluated prey quality based on flesh/shell ratio and shell hardness based on the break force of shells. The flesh/shell ratio was calculated using AFDM and DM_shell_ intake by each individual (AFDM/DM_shell_). A higher flesh/shell ratio (van Gils, de Rooij, et al., 2005) indicates higher prey quality. A fixed digital force gauge (HP‐20 and HP‐300, Yueqing Ai Li Instrument, China) was used to measure the break force (N) of four major prey of Great Knots (*P. laevis*, *M. veneriformis*, *M. iridescens*, and *U. thomasi*) (Bom et al., 2018; Yang et al., 2013). We regressed break force on prey size trying polynomial, e‐exponential, logarithmic, and power regression. Models with the largest Pearson correlation coefficient were selected.

### 2.4 Gizzard size

Some Great Knots drowned accidentally in fishing nets set on the intertidal mudflat. In April–May 2011–2012 and 2016–2017, a total of 48 dead birds (22 and 26, respectively) were collected and used opportunistically for the study. The birds were collected, the feathers dried with a hairdryer and weighed. Carcasses were sealed in airtight plastic bags and stored at –20°C. In laboratory, carcasses were dissected following the procedures of Piersma et al. (1999). The fresh mass of the gizzard was weighed to the nearest 0.1 g. The contents of the intestines and gizzard were washed separately through a 0.3 mm sieve and then dried at 60°C for 72 hr. The fragments were separated and identified. The shell fragments and the columella of *U. thomasi* in intestinal and gizzard contents were weighed to the nearest 0.1 mg.

### 2.5 Data analysis

We used Ivlev's electivity index (*E_i_*, Ivlev, 1961) to quantify the prey selection by Great Knots: *E_i_* = (*r_i_* – *p_i_*)/(*r_i_* + *p_i_*), where *r_i_* is the percentage of prey species *i* in the diet (number of species/total number of preys in the diet × 100), *p_i_* is the percentage of this species on the mudflat. *E_i_* ranges from −1 to 1 interval, values from −1 to 0 meaning negative selection, values from 0 to 1 meaning positive selection, and 0 meaning no selection.

Linear models controlling the effect of date were used to compare gizzard size (dry mass, g) between the period before (2011 and 2012 combined) and after food changes (2016 and 2017 combined). We calculated the changes of shell processing capacity based on the quadratic relationship between gizzard mass and shell processing capacity (van Gils, Piersma, Dekinga, & Battley, 2006). The percentage by weight of columella of *U. thomasi* in feces, pellets, intestines, and gizzard were compared using analysis of variance (ANOVA) followed by Fisher's least significant difference (LSD). Differences in intake rate of AFDM, DM_shell_, and prey quality between the two periods were also compared using ANOVA followed by LSD.

Logarithmic transformation was used when the data were not normally distributed. When modeling the functional response between intake rate and prey density, we took account of the variation in prey quality using a modified form of Holling's disk equation: IR* *= *a × N*/(1 + *a *× *N × h*/*Q*), where IR is the average intake rate (mg AFDM/s) for each year, *N* is the average prey density (ind/m^2^) for each year, and *Q* is the average prey quality (AFDM/DM_shell_) for each year. We estimated *a* (searching efficiency) and *h* (prey handling time) using the nonlinear least square (nls) function in R version 3.5.2. By multiplying handling time *h* by the inverse of prey quality *Q*, we included time lost to digestion in the standard type II functional response model (similar to eq. 5 in Jeschke, Kopp, & Tollrian, 2002). The significance level was set at 0.05. Statistical analyses were carried out in SPSS 20.0 unless referenced otherwise.

## 3 RESULTS

**Figure 3:**
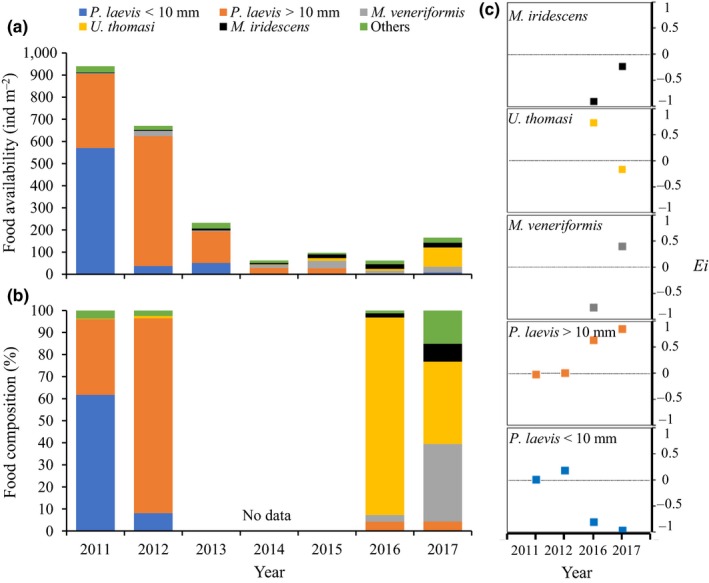
(a) Food availability (ind/m^2^) and (b) diet composition (percentage of individual prey in feces and pellets combined) of Great Knots from 2011 to 2017; (c) Electivity index (*E_i_*) of Great Knots for different prey in 2011, 2012, 2016, and 2017. *E_i_* in the interval of [–1, 0) means negative selection, (0, 1] positive selection, and 0 no selection

Prey available to Great Knots declined dramatically from 2011 to 2017. The density of small *P. laevis* (<10 mm) declined by 98.5%, *P. laevis* (>10 mm) declined by 99.8%, while densities of the gastropod *U. thomasi* increased 57 times and the bivalve *M. iridescens* increased seven times, respectively (Figure 3a). The main prey of Great Knots in 2011 was *P. laevis* (of total prey consumed 62% for small sized and 34% for large sized) and in 2012 (8% for small sized and 88% for large sized). In 2016, the main prey was *U. thomasi* (89.6%), and in 2017 *U. thomasi* (37%) and *M. veneriformis* (35%) (Figure 3b). In 2011 and 2012, Great Knots exhibited positive selection for small *P. laevis*, while they selected large *P. laevis* in 2016 and 2017 (Figure 3c). In 2016, Great Knots selected *U. thomasi *while rejecting *M. veneriformis,* but in 2017, they selected *M. veneriformis* (Figure 3c). Great Knots exhibited negative selection for small *P. laevis* in 2016 and 2017 (Figure 3c).

Break force of the four main prey species increased with shell length (Supporting Information Figure S2). The break forces of modal prey size were 4.0 N (*P. laevis*, 7.86 mm) in 2011, 12.0 N (*P. laevis*, 12.91 mm) in 2012, 45.2 N (*U. thomasi*, 11.61 mm) in 2016, and 35.4 N (*U. thomasi*, 10.37 mm) in 2017 (Supporting Information Figure S2). The break force required to crush the main prey in 2016 and 2017 was 9–11 times greater than that in 2011 and 3–4 times greater than that in 2012.

After controlling the effect of date, gizzard mass significantly increased (*F = *8.72, *p = *0.005) after the diet shift (a 15% increase from 7.86 ± 0.31 g in 2011–2012 to 9.43 ± 0.41 g in 2016–2017, Figure 4). This would equal a 32% increase in shell processing capacity.

**Figure 4:**
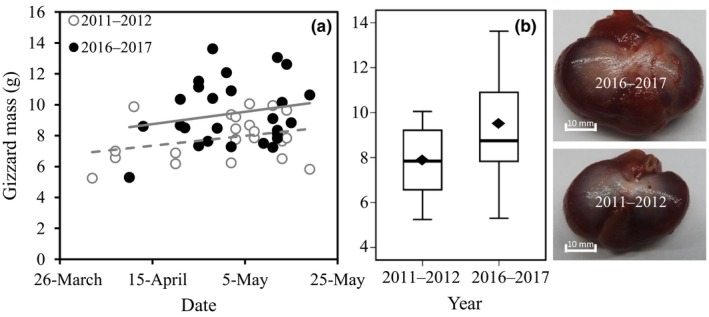
Comparison of gizzard mass of Great Knots in 2011–2012 (*n* = 22, open circles and dashed line) and 2016–2017 (*n* = 27, filled circles and solid line). (a) The equation in 2011–2012: Gizzard mass = 7.20 + 0.04 × capture date (Julian day: 1 Mar = 1)–1.53; in 2016–2017: Gizzard mass = 7.20 + 0.04 × capture date. (b) Gizzard mass was significantly different (*F* = 8.716, *p* = 0.005) between the two periods. Diamonds show the mean value of gizzard mass, horizontal lines show the median, the upper and lower edges of the box plots represent the first and the third quartiles, bars represent the 95% confidence interval. (c) Photographs of gizzards in 2011–2012 (bottom) and in 2016–2017 (top)

**Figure 5:**
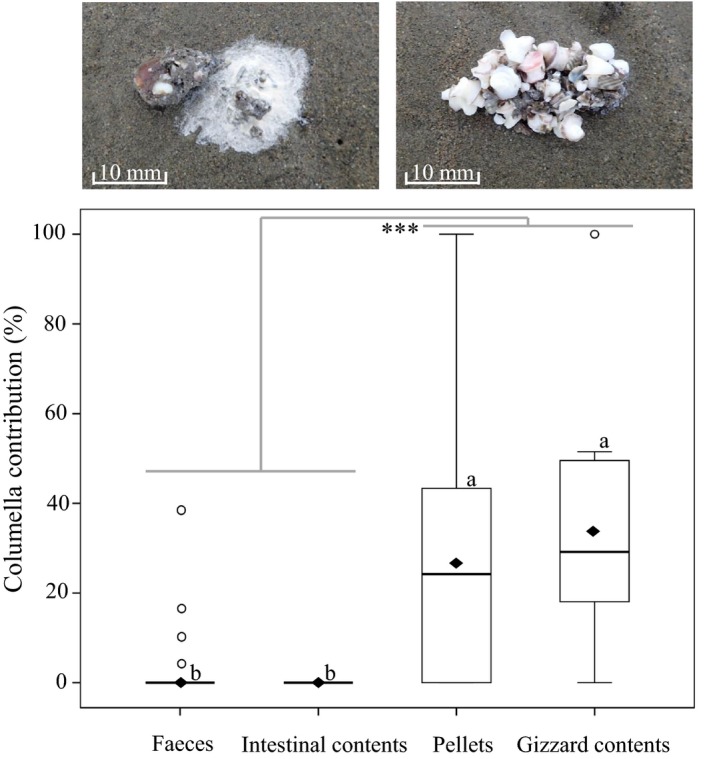
The weight percentage of columella remains in feces, intestinal contents, pellets, and gizzard contents. The three asterisks refer to a significant difference in the columella percent (%) between the combination of faces and intestinal contents and the combination of pellets and gizzard contents (*F = *40.88, *p* < 0.001). Diamonds show the mean value, horizontal lines show the median, the upper and lower edges of the box plots represent the first and third quartiles, the bars represent the 95% confidence interval. Open circles indicate outliers. The top‐left photograph shows feces of a Great Knot on the mudflat and the top‐right a pellet. The letters a and b denote significant differences detected by LSD test

The consumption of hard‐shelled mollusks in 2016–2017 was accompanied by a greater reliance on regurgitation (rather than intestinal evacuation as feces) to excrete unbreakable parts of prey such as the columella of *U. thomasi* (Figure 1). The weight percentages of columella in feces, intestinal contents, pellets, and gizzard contents were 0.71 ± 0.44, 0.00 ± 0.00, 27.18 ± 1.96, and 35.47 ± 5.34, respectively (Figure 5). There was no significant difference in the weight percentage of columella in feces and intestinal contents, and in pellets and gizzard contents (*p* > 0.05 for both), respectively (Figure 5). However, the weight percentage of columella in feces and intestinal contents was significantly lower than that in pellets and gizzard contents (*p < *0.001, Figure 5), suggesting that—unlike other parts of the shell—the columella was not passed through the entire digestive tract.

**Figure 6:**
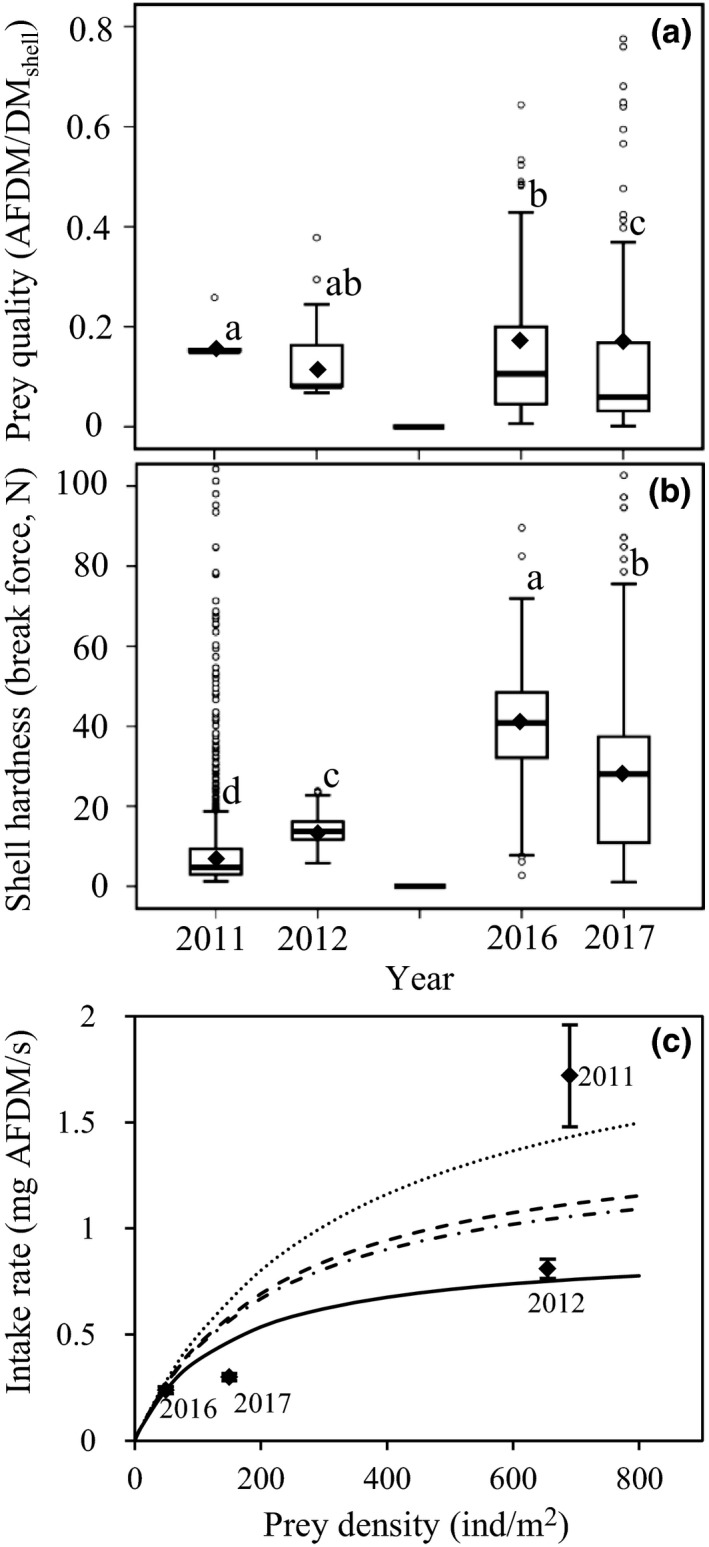
(a) Quality (AFDM/DM_shell_), and (b) shell hardness (break force, N) of prey of Great Knots in 2011, 2012, 2016, and 2017, and (c) the relationship between intake rate of ash‐free dry mass (AFDM/s) and prey density. Diamonds show the mean, horizontal lines show the median, the upper and lower edges of the box plots represent the first and third quartiles, and the bars represent the 95% confidence interval (a, b) or standard error (c). The open circles indicate outliers (a, b). Different letters denote significant differences detected by LSD test (a, b). The curves in (c) (the dotted line, dashed lines, dotted‐dashed line, and solid line represent 2011, 2012, 2016, and 2017, respectively) show the relationships between intake rate of ash‐free dry mass (AFDM/s) and prey density for different prey qualities expressed as: IR = a × *N*/(1 + *a* × *N* × *h*/*Q*), where IR, *N*, and *Q* are, respectively, the average intake rate (mg AFDM/s), prey density (ind/m^2^), and average prey quality (AFDM/DM_shell_) for each year; *a* (searching efficiency) = 6.5 × 10^−3^ m^2^/s, *h* (prey handling time) = 7.3 × 10^−2^ s/mg DM_shell_. At a given prey density, intake rate increases with prey quality

After the shift from *P. laevis *in 2011–2012 to a predominantly gastropod diet in 2016–2017, the intake rates expressed as AFDM/s and DM_shell_/s significantly decreased (*F = *91.73, *p < *0.001 and *F = *36.94, *p < *0.001, respectively) (Supporting Information Figure S1a,b). The intake rate of AFDM decreased by 86%, and the intake rate of DM_shell_ decreased by 77% (Supporting Information Figure S1a,b). The prey quality significantly differed among the 4 years (*F = *8.56, *p < *0.001), with the highest in 2011 and the lowest in 2017. There was no significant difference in prey quality in 2011 and 2012. However, that prey quality in 2016 and 2017 was significantly lower than in 2011 (*p < *0.05) and prey quality in 2017 was also significantly lower than in 2012 (*p < *0.05, Figure 6a), suggest a serious decrease in prey quality in the course of this study. Similarly, shell hardness of prey significantly differed among the 4 years (*F = *456.54, *p < *0.001), with the hardest in 2016 and the softest in 2011. Shell hardness in 2016 was 4.0 and 2.7‐fold harder than that in 2011 and 2012, respectively; shell hardness in 2017 was 2.8 and 2.0‐fold harder than that in 2011 and 2012, respectively (Figure 6b).

The functional response relationship between intake rate and prey density for the year‐specific prey qualities (Figure 6c) showed that the difference in intake rates between the two periods was explained by changes in both prey density and prey quality. The latter was especially evident from the comparison between 2011, when small *P. laevis *were abundant and large *P. laevis* were scarce, and 2012, when the reverse situation occurred.

## 4 DISCUSSION

This study yields an interesting example of the morphological and digestive adjustments by which animals cope with environmental change (Abrahms et al., 2018; Colles, Liow, & Prinzing, 2009; van Gils, Battley, et al., 2005). After a dramatic food decline at a staging site, Great Knots changed the composition of their diet composition and prey selection to include prey types that were physically more difficult to process. They could do so because they simultaneously adjusted both the features of the digestive organ (gizzard mass) and the way of dealing with indigestible matter (voiding the hardest shell fragments through regurgitates rather than as feces).

The decline of food availability was mainly due to an almost complete loss of *P. laevis*. *P. laevis* occurred at high densities in 2011 and 2012, accounting for more than 95% of the total macrobenthos (Choi et al., 2017; Zhang et al., 2018). The small *P. laevis* are relatively easy to crush in the gizzard (Yang et al., 2013), thus birds exhibited a high processing rate and thus a high intake rate. Indeed, Great Knots exhibited positive selection for small *P. laevis* in 2011 and 2012. By 2016 and 2017, small *P. laevis* had all but disappeared and *M. iridescens* only occurred at low densities (Figure 3a). However, there were still a few large *P. laevis* available, which were selected by Great Knots (Figure 3c).

According to symmorphic design rules, organs should grow to a size to satisfy but not exceed the requirements (Taylor & Weibel, 1981). Digestive organs have a high energy cost per unit mass (Rolfe & Brown, 1997; Scott & Evans, 1992), and the amount and metabolic cost can increase basal metabolic rate (BMR) (McKechnie, 2008; Piersma, 2002; Piersma et al., 1996; Vézina, Love, Lessard, & Williams, 2009). This is suggested to be one of the reasons that migratory birds ready for take‐off on long‐distance flights avoid oversized digestive organs (Piersma & Gill, 1998; van Gils, Battley, et al., 2005; Yang et al., 2013). However, this is also a function of ecological context as Red Knots, when forced to eat hard‐shelled food generally enlarge their gizzard, to reduce it when soft food is available (Dekinga et al., 2001; van Gils et al., 2003). At YLJ, the prey quality decreased from 2011–2012 to 2016–2017, which means birds would need to process more shell material for similar amounts of energy gained. However, the shell of prey consumed in 2016 and 2017 required larger break force to be crushed than those in 2011 and 2012 (Figure 6b). The larger the break force, the longer the processing time (Yang et al., 2013). Thus, Great Knots feeding on hard‐shelled mollusks will be digestively constrained by the amount of prey processed per unit time (Piersma, Koolhaas, & Dekinga, 1993; van Gils, Battley, et al., 2005; van Gils et al., 2003). As a consequence, the AFDM intake rate of Great Knots not only decreased with prey density, but also decreased with prey quality and shell hardness (Figure 6c). Great Knots at YLJ increased gizzard mass by 15% which, if similar to Red Knots, would have resulted in an increase of the shell processing rate by 32% (van Gils et al., 2006). This, however, will not be sufficient to compensate for the overall reduction in food availability and quality.

Shorebirds usually evacuate crushed shell material from the gut as feces (Battley & Piersma, 2005). The regurgitation of pellets, which occurs in Tringids but has not often been reported in Calidrid sandpipers (Dekinga & Piersma, 1993; Fedrizzi, Carlos, & Campos, 2016; Worrall, 1984), coincided with the strongly increased break forces required for ingested prey in 2016 and 2017. It appears that Great Knots were unable to crush the extremely hard calcified columella of *U. thomasi*. The potential risk of the columella, with its pointed apex and relatively sharp whorls, damaging the digestive tract (see Piersma et al., 1993; van Gils, Battley, et al., 2005; Yang et al., 2013; pers. obs.), may explain the change from a one‐way stream to a two‐way stream for ballast evacuation (see Figure 1).

The long‐term AFDM intake rate in 2016 and 2017 was only 14%–30% of that in 2011 and 2012 (Figure 6a). As a consequence, to achieve the daily body mass gain at YLJ necessary to migrate, Great Knots would need to forage for 13 – 16 hr per day in 2016–2017 rather than less than five hours in 2011–2012 (Supporting Information Appendix S1). The mudflats at YLJ are exposed for an average of 18.6 hr a day during spring tides and 21.0 hr a day during neap tides (C.Y. Choi et al., unpublished data). However, Great Knots eating very hard‐shelled *U. thomasi *were observed to take long pauses of over one hour (pers. obs., see van Gils, Battley, et al., 2005). We interpret this as time necessary for digestion and/or the preparation and voidance of regurgitates. It thus appears likely that birds were rather challenged to have enough foraging time to satisfy the energy requirements, especially at spring tides.

Staging numbers of Great Knots at YLJ declined by a third between 2011/2012 and 2016/2017 (Zhang et al., 2018), implying that some birds were not able to fully compensate for the severe reduction in both prey quantity and quality. Considering the effects on survival of the dramatic losses and degradation of intertidal habitats in the Yellow Sea (Piersma et al., 2016), migrating Great Knots appear to be short of alternative high‐quality staging habitats. The decrease in body mass gain rates of Great Knots at the study site could well lead to cause downstream effects on survival and breeding success (Piersma et al., 2016; Senner, Conklin, & Piersma, 2015).The long‐term studies on birds and their food conditions at the staging and nonbreeding sites which spawned this contribution, seem critical if we are serious in trying to understand the causes of decline and to responsively manage the key habitats of endangered species such as Great Knots.

## CONFLICT OF INTEREST

None declared.

## AUTHORS’ CONTRIBUTIONS

Z.M, T.P., J.A.v.G., and S.D.Z. conceived the ideas and designed the experiment; S.D.Z., C.Y.C., H.B.P., D.S.M., Q.Q.B., T.T.Z, and Y.C.C. collected the data in the field; S.D.Z, C.Y.C., and W.L.L processed the samples and recordings with help from H.B.P. and Y.C.C.; S.D.Z. analyzed the data; S.D.Z., T.P., and Z.M. led the writing of the manuscript. All authors contributed to the drafts and approved the final version.

## Data Availability

Relevant data will be available via Dryad: https://doi.org/10.5061/dryad.9021d2v.
