# Supplementary figures and images for: Morphological and digestive adjustments buffer performance: How staging shorebirds cope with severe food declines
Source: Ecol Evol. 2019 Mar 12;9(7):3868–78. doi: 10.1002/ece3.5013 (PMC6468082; doi:10.1002/ece3.5013)

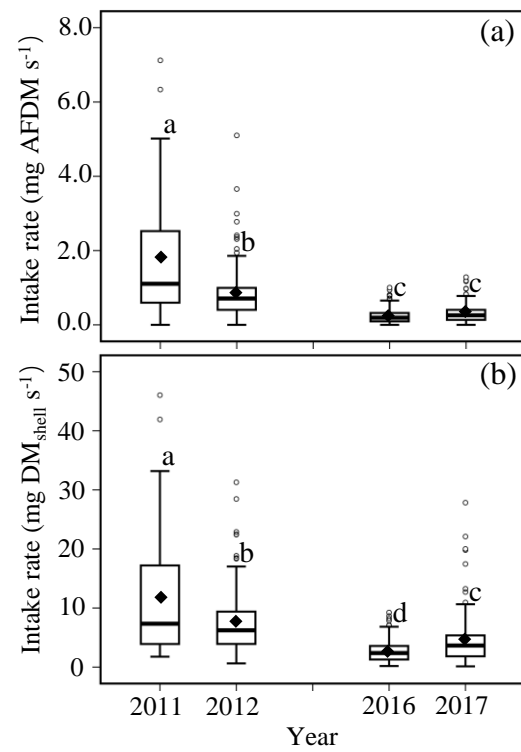

Supplement: Supplementary file 1 [file ECE3-9-3868-s001.pdf]

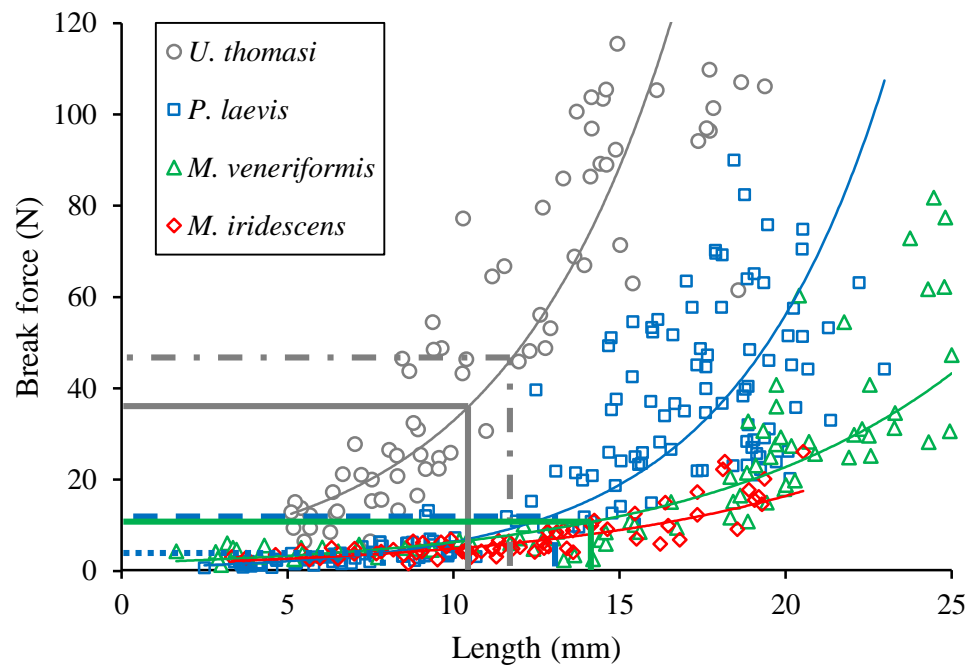

Supplement: Supplementary file 2 [file ECE3-9-3868-s002.pdf]
