# Supplementary material for: Morphological and digestive adjustments buffer performance: How staging shorebirds cope with severe food declines
Source: Ecol Evol. 2019 Mar 12;9(7):3868–78. doi: 10.1002/ece3.5013 (PMC6468082; doi:10.1002/ece3.5013)
Supplement: Supplementary file 3 [file ECE3-9-3868-s003.docx]

**APPENDIX**

A shorebird the size of a Great Knot has a daily energy expenditure (DEE) of 1.8 basal metabolic rate (BMR) (Piersma, Drent, & Wiersma, 1991) or 2.81W when allowing for thermostatic costs, maintenance of body processes and activity (Tulp & de Goeij, 1994). When the birds were engaged in energetically cheap activities such as sleeping, standing, and preening, we assumed that the DEE decreased to 1.2 BMR (Tulp & de Goeij, 1994) or 1.87 W for periods of low activity period. Considering the birds spent 16 hours (70%) active and 8 hours (30%) at rest (Tulp & de Goeij, 1994),

daily energy expenditure (DEE) is:

DEE = DEE_active_ + DEE_rest_ = (0.7 × 1.8 BMR × 16 × 3600) + (0.3 × 1.2 BMR × 16 × 3600 +1.2 BMR × 8 × 3600) = 199 KJ day^-1^ (A1) (Tulp & de Goeij, 1994)

With a fuel deposition efficiency of 0.88 (Kersten & Piersma, 1987), the daily surplus is:

Daily surplus = (fat mass gain × energy value (EV) of fat + protein and glycogen mass gain × EV of protein and glycogen)/ deposition efficiency = (1.55 × 58.97% × 38+1.55 × 41.03% × 5.5)/0.88= 33.64 KJ day^-1^ (A2)

Here, the EV of fat is taken as 38 kJ per g fat, that of protein and glycogen is taken 5.5 kJ per g protein and glycogen wet mass (Piersma & Lindström, 1997). Great Knots must attain a body mass of at least 220g to enable them to fly directly from YLJ to their breeding grounds (Ma *et al*., 2013). This means that, taking into account the average stopover duration of 31 days at YLJ (Ma *et al*., 2013). A bird must gain at least 1.55g of body weight per day (Ma *et al*., 2013). Based on body composition analysis of Great Knot at YLJ in the spring, we know that the daily dry fat mass accumulation accounts for 56.16% of body weight accumulated (Hua *et al*., 2014). Fat is usually deposited with 5% water (Piersma & Lindström, 1997), so the daily fat mass accumulation accounts for 59% of body weight accumulation and protein and glycogen both wet mass accounts for 41%.

Daily energy expenditure + Daily surplus = Daily net energy intake rate = 199 + 33.64 = 232.64 kJ day^-1^ (A3)

Therefore, Great Knots at YLJ must be able to reach the daily net energy intake rate if they are to lay down reserves for migration.

Daily net energy intake rate (NEI) was calculated from intake rate (IR), using the equation:

NEI = IR × EV × AE × length of working day (A4)

Here, EV represents the energetic value of 1 g AFDM, which was assumed to be 22 kJ (Chambers & Milne, 1975; Dare & Edwards, 1975; Beukema & de Bruin, 1977) and AE is the assimilation efficiency which is close to 0.75 (Kersten & Piersma, 1987).

Therefore, the length of the working day was calculated from the equation below:

Length of the working day = NEI × 1000 / (3600 × IR × EV × AE) (A5)

The length of working day calculated for each northward migration season are 2.2, 4.8, 16 and 13 hours in 2011, 2012, 2016 and 2017, respectively.

**References**

Beukema, J. J., & de Bruin, W. (1977). Seasonal changes in dry weight and chemical composition of the soft parts of the tellinid bivalve *Macoma balthica* in the Dutch Wadden Sea. *Netherlands Journal of Sea Research*, 11, 42–55. https://doi.org/10.1016/0077-7579(77)90020-5

Chambers, M. R., & Milne, H. (1975). The production of *Macoma balthica* (L.) in the Ythan estuary. *Estuarine and Coastal Marine Science*, 3, 443–455. https://doi.org/10.1016/0302-3524(75)90044-4

Dare, P. J., & Edwards, D. B. (1975). Seasonal changes in flesh weight and biochemical composition of mussels (*Mytilus edulis* L.) in the Conwy estuary, north Wales. *Journal of Experimental Marine Biology and Ecology*, 18, 89–97. https://doi.org/10.1016/0022-0981(75)90066-0

Hua, N. (2014). Fuel Deposition of Shorebirds at Stopping Sites in the Yellow Sea during Spring Migration. PhD thesis, Fudan University, China.

Kersten, M., & Piersma, T. (1987). High levels of energy expenditure in shorebirds; metabolic adaptations to an energetically expensive way of life. *Ardea*, 75, 175–187. https://doi.org/10.5253/arde.v75.p175

Ma, Z. J., Hua, N., Peng, H. B., Choi, C., Battley, P. F., Zhou, Q. Y., … Xue, W. (2013). Differentiating between stopover and staging sites: functions of the southern and northern Yellow Sea for long–distance migratory shorebirds. *Journal of Avian Biology*, 44, 504–512. https://doi.org/10.1111/j.1600-048X.2013.00213.x

Piersma, T., & Lindström, Å. (1997). Rapid reversible changes in organ size as a component of adaptive behaviour. *Trends in Ecology & Evolution*, 12, 134–138. https://doi.org/10.1016/S0169-5347(97)01003-3

Piersma, T., Drent, R., & Wiersma, P. (1991). Temperate versus tropical wintering in the world’s northernmost breeder, the knot: metabolic scope and resource restrict subspecific options. *Acta XX Congressus Internationalis Ornithologici*, 761–772.

Tulp, I., & de Goeij, P. (1994). Evaluating wader habitats in Roebuck Bay (North–western Australia) as a springboard for Northbound migration in waders, with a focus on Great Knots. *Emu*, 94, 78–95. https://doi.org/10.1071/MU9940078

Figure S1. Intake rate of (a) ash free dry mass (AFDM) and (b) dry mass of shell (DM_shell_) of Great Knots in terms of average flesh/shell ratio. Diamonds show the mean, horizontal lines show the median, the upper and lower edges of the box plots represent the first and third quartiles, bars represent the 95% confidence interval, and open circles indicate outliers. Different letters denote significant differences detected by LSD test (a, b).

Figure S2. Break force (y) as a function of individual shell length (x) in four major prey species: the gastropod *Umbonium thomasi* (y = 4.56×e^0.20×x^, *R²* = 0.84, n = 93), and bivalves *Potamocorbula laevis* (y = 0.72×e^0.22×x^, *R²* = 0.87, n = 165), *Mactra veneriformis* (y = 1.74×e^0.13×x^, *R²* = 0.79, n = 80) and *Moerella iridescens* (y = 1.45×e^0.12×x^, *R²* = 0.72, n = 67), respectively. The dotted line, dashed lines, dotted-dashed line and solid line represent break force of the prey species (different colour) for the shell length most frequently eaten in 2011, 2012, 2016 and 2017, respectively.

Table S1. Relationship between ash free dry mass of flesh (AFDM, g) and shell length (L, mm) for bivalves, width of the last whorl (W, mm) for *Umbonium thomasi*, and shell height (H, mm) for other gastropods.

| Species | Class | Equation | *R^2^* | *N* | Year |
| --- | --- | --- | --- | --- | --- |
| *Potamocorbula laevis* | Bivalvia | AFDM = 10^-5^×L^2.95^ | 0.97 | 135 | 2011 |
| *Potamocorbula laevis* | Bivalvia | AFDM = 2×10^-5^×L^2.60^ | 0.88 | 101 | 2012 |
| *Potamocorbula laevis* | Bivalvia | AFDM = 5×10^-6^×L^3.01^ | 0.95 | 34 | 2013–2017 |
| *Mactra veneriformis* | Bivalvia | AFDM = 2×10^-6^×L^3.43^ | 0.96 | 99 | 2011–2017 |
| *Meretrix meretrix* | Bivalvia | AFDM = 10^-6^×L^3.52^ | 0.94 | 20 | 2011–2017 |
| *Moerella iridescens* | Bivalvia | AFDM = 8×10^-6^×L^2.59^ | 0.87 | 35 | 2011–2017 |
| *Anadara kagoshimensis* | Bivalve | AFDM = 7×10^-6^×L^3.14^ | 0.96 | 12 | 2011–2017 |
| *Ruditapes philippinarum* | Bivalvia | AFDM = 4×10^-6^×L^3.13^ | 0.90 | 11 | 2011–2017 |
| *Dosinia laminata* | Bivalvia | AFDM = 2×10^-6^×L^3.41^ | 0.99 | 19 | 2011–2017 |
| *Mactra chinensis* | Bivalvia | AFDM = 9×10^-6^×L^2.99^ | 0.95 | 5 | 2011–2017 |
| *Sinonovacula constricta* | Bivalvia | AFDM = 3×10^-6^×L^3.12^ | 0.88 | 6 | 2011–2017 |
| *Laternula* sp*.* | Bivalvia | AFDM = 10^-6^×L^3.26^ | 0.92 | 10 | 2011–2017 |
| *Umbonium thomasi* | Gastropoda | AFDM = 7×10^-6^×W^3.32^ | 0.98 | 37 | 2011–2017 |
| *Nassarius festivus* | Gastropoda | AFDM = 4×10^-6^×H^3.20^ | 0.91 | 33 | 2011–2017 |
| *Nassarius variciferus* | Gastropoda | AFDM = 10^-5^×H^2.88^ | 0.97 | 13 | 2011–2017 |
| *Neverita didyma* | Gastropoda | AFDM = 2×10^-5^×W^3.23^ | 0.99 | 14 | 2011–2017 |
| *Lunatia gilva* | Gastropoda | AFDM = 10^-6^×H^4.28^ | 0.96 | 6 | 2011–2017 |
| *Bullacta exarata* | Gastropoda | AFDM = 10^-5^×L^3.21^ | 0.98 | 30 | 2011–2017 |
| *Lingula anatina* | Brachiopod | AFDM = 5×10^-6^×L^3.01^ | 0.95 | 27 | 2011–2017 |

Table S2. Relationship between shell dry mass (DM_shell_, g) and shell length (L, mm) for bivalves, width of the last whorl (W, mm) for *Umbonium thomasi*, and shell height (H, mm) for other gastropods.

| Species | Class | Equation | *R^2^* | *N* |
| --- | --- | --- | --- | --- |
| *Potamocorbula laevis* | Bivalvia | DM_shell_ = 2×10^-5^×L^3.38^ | 0.93 | 34 |
| *Mactra veneriformis* | Bivalvia | DM_shell_ = 6×10^-5^×L^3.12^ | 0.99 | 99 |
| *Moerella iridescens* | Bivalvia | DM_shell_ = 4×10^-5^×L^2.92^ | 0.99 | 35 |
| *Anadara kagoshimensis* | Bivalvia | DM_shell_ = 10^-4^×L^3.01^ | 0.99 | 12 |
| *Ruditapes philippinarum* | Bivalvia | DM_shell_ = 7×10^-5^×L^3.06^ | 0.99 | 11 |
| *Meretrix meretrix* | Bivalvia | DM_shell_ = 7×10^-5^×L^3.18^ | 0.99 | 20 |
| *Dosinia laminata* | Bivalvia | DM_shell_ = 6×10^-5^×L^3.15^ | 0.98 | 19 |
| *Mactra chinensis* | Bivalvia | DM_shell_ = 10^-4^×L^2.62^ | 0.99 | 5 |
| *Sinonovacula constricta* | Bivalvia | DM_shell_ = 4×10^-5^×L^2.65^ | 0.97 | 6 |
| *Laternula* sp*.* | Bivalvia | DM_shell_ = 10^-6^×L^4.08^ | 0.90 | 10 |
| *Umbonium thomasi* | Gastropoda | DM_shell_ = 7×10^-5^×W^3.29^ | 0.99 | 37 |
| *Bullacta ecarata* | Gastropoda | DM_shell_ = 10^-5^×L^3.03^ | 0.94 | 30 |
| *Nassarius festivus* | Gastropoda | DM_shell_ = 2×10^-4^×H^2.69^ | 0.97 | 33 |
| *Nassarius variciferus* | Gastropoda | DM_shell_ = 3×10^-4^×H^2.47^ | 0.97 | 13 |
| *Neverita didyma* | Gastropoda | DM_shell_ = 3×10^-4^×W^2.70^ | 0.99 | 14 |
| *Lunatia gilva* | Gastropoda | DM_shell_ = 5×10^-5^×H^3.16^ | 0.98 | 6 |
| *Lingula anatina* | Brachiopod | DM_shell_ = 8×10^-7^×L^3.70^ | 0.93 | 27 |

Table S3. Relationship between hinge height (H, mm) and shell length (L, mm) for bivalve species, and between width of the last columella (S, mm) and width of the last whorl (W, mm) for *Umbonium thomasi* in the diet of Great Knots.

| Mollusc species | Equation | *R^2^* | *N* |
| --- | --- | --- | --- |
| *Potamocorbula laevis* (left shell) | L = 12.57×H + 5.05 | 0.85 | 19 |
| *Mactra veneriformis* (left shell) | L = 11.60×H - 2.67 | 0.84 | 88 |
| *Umbonium thomasi* | W = 3.35×S + 1.89 | 0.94 | 61 |
| *Moerella iridescens* (left shell) | L = 17.40×H + 2.39 | 0.65 | 48 |
| *Ruditapes philippinarum* (left shell) | L = 14.35×H + 3.99 | 0.91 | 11 |
